# Photoacoustic Imaging in Inflammatory Orthopedic Diseases: Progress toward Precise Diagnostics and Predictive Regulation

**DOI:** 10.1002/advs.202412745

**Published:** 2025-02-28

**Authors:** Mengyi Huang, Haoyu Yu, Rongyao Gao, Yuxin Liu, Xuhui Zhou, Limin Fu, Jing Zhou, Luoyuan Li

**Affiliations:** ^1^ The Eighth Affiliated Hospital Sun Yat‐sen University Shenzhen Guangdong 518033 P. R. China; ^2^ Department of Chemistry Renmin University of China Beijing 100872 P. R. China; ^3^ Department of Chemistry University of Chicago 5735 S Ellis Ave Chicago IL 60635 USA; ^4^ Department of Chemistry Capital Normal University Beijing 100048 P. R. China

**Keywords:** inflammatory orthopedic diseases, photoacoustic imaging, precise diagnostic, stimuli‐responsive nanoprobes

## Abstract

With the intensification of aging issues, inflammatory orthopedic diseases almost occur in the majority of elderly people, which is becoming increasingly severe. Photoacoustic imaging (PAI) is a non‐invasive visualization technique for a clear diagnosis of the inflammation areas through detecting acoustic signals generated by the laser irradiation. The combination of “light input” and “acoustic output” provides unprecedented scalability as well as high penetration depth and resolution. This new imaging technology can also present more anatomical information and feedback status of inflammatory activity for the orthopedic diseases. Especially in inflammation imaging, this technology can effectively supplement current clinical imaging methods in diagnosis, staging, and monitoring of pathophysiological processes. With the rapid development of these new technologies, the goals of precise diagnosis, predictive regulation, and ultimately personalized treatment strategies are becoming increasingly realistic. Herein, this article introduces various orthopedic inflammations and related imaging technology applications. It covers the types of PA nanoprobes and their research progress in orthopedic inflammation, as well as the potential applications of PAI in various aspects. The review also discusses the recent researches and emerging translational applications of PAI in orthopedic inflammation, as well as the prospects and future development challenges of clinical transformation.

## Graphical abstract

With the intensification of aging issues, inflammatory orthopedic diseases almost occurs in every elderly people, which are becoming increasingly severe. Persistent inflammation of joints often leads to irreversible tissue damage and loss of organ function, while early diagnosis and timely intervention are crucial for reducing the personal and social burdens. Photoacoustic imaging (PAI) is a non‐invasive visualization technique for clear diagnosis of inflammation in the bone joint areas by combination of “light input” and “sound output”, which provides unprecedented scalability as well as high penetration depth and resolution. This article reviews the progress of orthopedic inflammations based on PAI toward precision medicine and the challenges on the diagnosis of inflammatory orthopedic diseases in clinical practice.

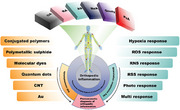

## 1 Introduction

**Figure 1:**
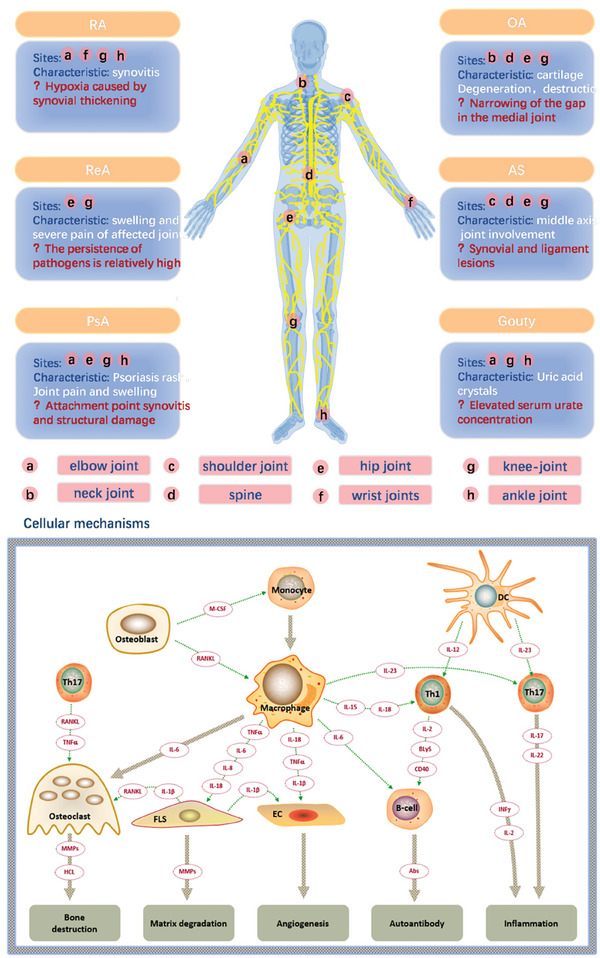
Classification of Orthopedic Inflammation. The multiple sites and characteristic manifestations of common or typical orthopedic inflammation, as well as related inevitable issues. RA, Rheumatoid Arthritis; OA, Osteoarthritis; ReA, Reactive arthritis; AS, Ankylosing Spondylitis; PsA, Psoriatic arthritis.

As the aging problem intensifies, the development of inflammatory orthopedic diseases becomes increasingly severe, which requires long‐term treatment and significant economic burden. Arthritis refers to an inflammatory disease that occurs in the joints and surrounding tissues of the human body caused by inflammation, infection, degeneration, trauma, or other factors, and can be divided into dozens of types. Clinical manifestations include joint redness, swelling, fever, pain, functional impairment, and joint deformities. In severe cases, it can lead to joint disability and affect the patient's quality of life. The etiology of arthritis is complex, mainly related to factors such as autoimmune reactions, infections, metabolic disorders, trauma, and degenerative diseases. Based on its etiology, arthritis can be classified into bone type, rheumatoid, ankylosing, reactive, gouty, rheumatic, purulent, etc (**Figure** **1**). With the increase of the proportion of people aged 60 and above, the incidence rate of arthritis is expected to continue to rise. The course of most arthritis is long and difficult to cure, making treatment very challenging. Therefore, achieving early detection, diagnosis, and treatment is beneficial for the prevention of inflammatory orthopedic diseases or improving the prognosis of patients.

Imaging examinations have always played an important role in assisting in the diagnosis and assessment of the severity of arthritis. Radiography (X‐ray) is widely available and low‐cost, but its imaging examination for radiation exposure has limited reproducibility in longitudinal studies. Magnetic resonance imaging (MRI) technology can evaluate joints as a whole organ through visualization of all tissues, but it shows high cost, long imaging time and low availability in some contraindications. Computed Tomography (CT) examination has advantages such as high anatomical resolution, and accurate description of focal cartilage defects. Its drawbacks include radiation exposure, lower soft tissue contrast agents, and insufficient visualization of bone marrow lesions. Ultrasound (US) technology has wide availability, fast imaging speed, radiation free, real‐time and multi plane evaluation, as well as color Doppler vascular distribution evaluation. However, it still has many limitations in terms of organizational specificity, contrast, and functional information. With the introduction of more tracers and new imaging tools, the arsenal of prevenient imaging modes has been expanded. Notably, Photoacoustic imaging (PAI) has attracted widespread attention, which can improve the organizational specificity and imaging contrast of functional and morphological information, allowing for in vivo evaluation of biochemical characteristics of individual disease status. **Table** **1** compares the advantages and disadvantages of various imaging techniques.

**Table 1:** The advantages and disadvantages of various imaging techniques.

Type	Advantage	Disadvantage
US	1. Low cost and real‐time imaging 2. No ionizing radiation	1. Relying on the skills of the operator 2. Low sensitivity to detecting changes in deep joints (hips, shoulders, hip joints) 3. Image clarity limit
X‐rays	1. Widely available 2. Cost‐effective	1. Only view two‐dimensional planar structures 2. Exposure to ionizing radiation 3. Low sensitivity to detecting early bone damage
CT	1. Multi plane reconstruction through computer processing 2. Convenient and rapid inspection	1. Radiation exposure 2. Insufficient visualization of bone marrow lesions 3. Unable to detect inflammatory activity
MRI	1. High sensitivity 2. No ionizing radiation	1. High cost and limited accessibility of equipment 2. The inspection lasts for a long time 3. Poor display of calcification and relatively poor display of bony structures
PAI	1. Multi scale and multi‐dimensional imaging 2. Detecting subtle structural changes in joint diseases 3. Non‐Invasive and provide functional information 4. Multi modal imaging capability 5. Flexible probe selection	1. Difficulty in extracting functional parameters 2. The widespread acceptance and popularization in clinical applications are limited

PAI is an emerging imaging method. When laser irradiates tissues, due to the scattering effect of biological tissues on light waves, light waves cannot be effectively focused, but electromagnetic wave energy can effectively enter the interior of tissues. Light absorbers (such as hemoglobin and melanin) within biological tissues absorb electromagnetic wave energy and convert it into thermal energy. The thermal expansion and contraction of the absorber emit acoustic signals, which are then received by highly sensitive detectors. Through signal processing and reconstruction, a photoacoustic image reflecting the internal structure and function of the tissue can be formed. Although PAI has a relatively short history in the field of biomedical imaging, its appeal as a non‐invasive, non‐ionizing, and multi parameter imaging method has led to rapid development in research.^[^ ^1^ ^]^ Most importantly, this new imaging method provides informative optical contrast, revealing details of anatomical, functional, molecular, and histological features.^[^ ^2^ ^]^

Endogenous molecules, such as deoxyhemoglobin, oxidized hemoglobin, collagen, lipids, and melanin, can achieve PAI in the near‐infrared (NIR) range due to their unique absorption characteristics. The clinical capabilities of this technology can be further enriched by specifically labeling target molecules or cell targeting exogenous probes or contrast agents.^[^ ^3^, ^4^ ^]^ PAI utilizes rich optical intrinsic and extrinsic contrast to provide in vivo information based on the light absorption characteristics of biological tissues.^[^ ^5^, ^6^ ^]^ The non‐invasive and tissue‐specific nature of PAI enables its widespread application in experimental and clinical environments without interfering with biological processes. Additionally, PAI can provide multi‐scale perspectives on inflammation processes, monitoring structures and functions at different anatomical positions, resolutions, and imaging depths. In summary, PAI is an emerging non‐invasive imaging method that has had a significant impact in revealing the pathophysiology of inflammation in preclinical and clinical settings.^[^ ^7^ ^]^ This review summarized the diagnostic advantages of photoacoustic imaging in common inflammatory orthopedic diseases and the future development of photoacoustic probes. Finally, this review discussed the emerging researches of photoacoustic imaging in orthopedic inflammation to achieve precise diagnosis and predictive regulation, as well as the prospects and future development challenges of clinical transformation.

## 2 Inflammatory Orthopedic Diseases

The following section discusses the clinical manifestations and related clinical imaging diagnoses of inflammatory orthopedic diseases. **Table** **2** provides an overview of the selected studies.

**Table 2:** Classification of orthopedic inflammation.

Type	Characteristic	Common affected areas	Common imaging examinations
Osteoarthritis	Joint swelling and deformity Tenderness and passive pain Joint friction sensation Morning stiffness and adhesion	More involvement of finger joints, knee joints, hip joints, spine, etc	X‐ray, MRI, ultrasound
Rheumatoid Arthritis	Highly sensitive to climate change rheumatoid nodules Joint deformities, swelling, pain Fever, chills, decreased appetite	Common involvement of proximal interphalangeal joint, metacarpophalangeal joint, and wrist joint	X‐ray, CT, MRI, ultrasound
Ankylosing Spondylitis	peripheral arthritis Spinal deformity Digestive system discomfort Iritis accounts for 10‐20% Pulmonary interstitial fibrosis is more common	The main lesion site is the spine, which affects the sacroiliac joint	X‐ray, CT, MRI
Reactive arthritis	Joint involvement varies in severity enthesitis Skin and mucosal lesions Fatigue, overall discomfort, muscle pain, and low fever	Mainly in the lower limbs, with the knee, ankle, and metatarsophalangeal joints being the most common	X‐ray, CT, MRI, ultrasound
Gouty	1. Acute gout affected joints and surrounding tissues present with redness, swelling, heat, and pain 2. Chronic gout presents with irregular, asymmetric swelling, and pain in the joints 3. Accompanied by renal damage	More common in the first metatarsophalangeal joint, dorsum of foot, ankle, knee, wrist, and other areas	X‐ray, CT, ultrasound
Psoriatic arthritis	1. peripheral arthritis 2. Psoriasis rash present 3. Nail damage 4. Tendon synovitis and attachment point inflammation 5. 25% to 70% PsA with involvement of the central axis joint	Invasion of distal interphalangeal joint	ultrasound

### 2.1 Osteoarthritis (OA)

OA is the most common degenerative joint disease. The pathological changes observed in OA joints include progressive loss and destruction of articular cartilage, thickening of subchondral bone, formation of osteophytes, varying degrees of synovial inflammation, degeneration of knee ligaments and ligaments, and hypertrophy of joint capsules. Degeneration of articular cartilage and increased mortality of chondrocytes are typical features of osteoarthritis.^[^ ^8^ ^]^ Possible reasons include mechanical and biological factors, which lead to imbalanced degradation and synthesis of chondrocytes, extracellular matrix, and subchondral bone.^[^ ^9^, ^10^ ^]^ In addition, these structural and functional changes are inherently accompanied by changes in optical properties at single or multiple wavelengths, which can potentially be sensed or mapped through PAI.

Compared to normal joints, OA joints undergo varying degrees of changes as OA progresses. In early OA, the surface contour of cartilage may appear slightly irregular, the superficial area of articular cartilage may undergo slight division, the subchondral bone plate may be thinner but also more porous, and some blood vessels may erode into avascular cartilage. In the late stage of osteoarthritis, the entire cartilage structure is completely destroyed, usually accompanied by subchondral bone sclerosis, narrowing of joint space, osteophyte formation, and more vascular erosion of cartilage.^[^ ^11^ ^]^

**Figure 2:**
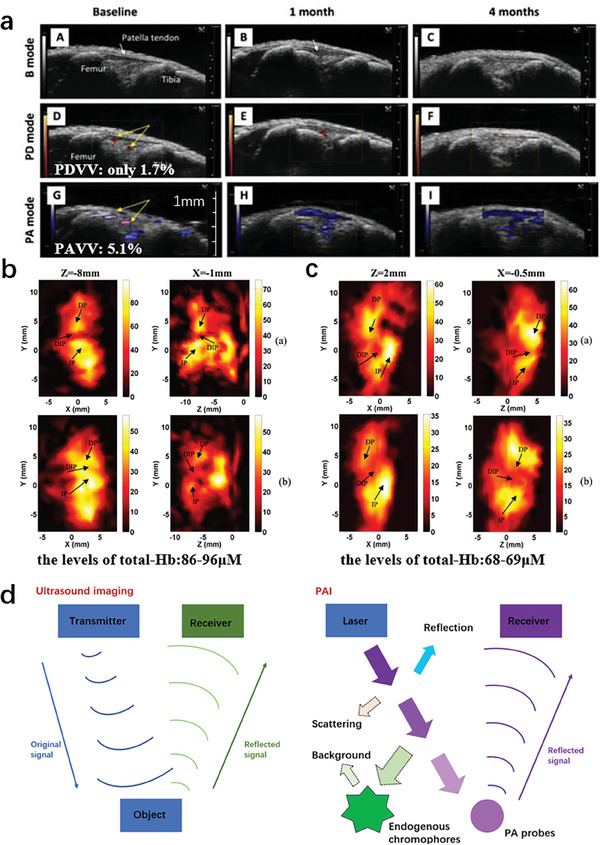
PA images of osteoarthritis. a) Representative B mode, PD, and PA images of knee joints from intact and DMM knee joints. DMM, a destabilization of the medial meniscus.^[^ ^12^ ^]^ b) Concentrations of oxy‐hemoglobin and deoxy‐hemoglobin in µM at coronal section (Z = −8 mm) and sagittal section (X = −1 mm) of examined finger joint from an OA patient. DP, distal phalanx; IP, intermediate phalanx; DIP, distal interphalangeal joint.^[^ ^13^ ^]^ c) Concentrations of oxy‐hemoglobin and deoxy‐hemoglobin in µM at coronal section (Z = 2 mm) and sagittal section (X = −0.5 mm) of examined finger joint from a healthy subject. DP, distal phalanx; IP, intermediate phalanx; DIP, distal interphalangeal joint.^[^ ^13^ ^]^ d) Schematic diagram of the principles of ultrasound imaging and PAI.

Synovial tissue hypoxia in a post‐traumatic osteoarthritis model was evaluated by using PAI and explored its correlation with the severity of OA. They used PAI technology to longitudinally monitor the three‐dimensional vascular structure and oxygenation level of synovial tissue in osteoarthritis mice induced by instability of the medial meniscus. PAI can achieve noninvasive imaging of synovial tissue. The characteristics of OA synovitis are increased angiogenesis and synovial tissue hypoxia; the latter is correlated with the severity of OA (**Figure** **2**a).^[^ ^12^ ^]^

Quantitative imaging of hemoglobin concentration and oxygen saturation in finger joints in vivo can be conducted and evaluated the feasibility of using 3D multispectral quantitative PA tomography (3D qPAT) to detect OA of the hand. The results indicate that the anatomical structure and quantitative chromophore concentrations (oxygenated hemoglobin and deoxyhemoglobin) of different joint tissues (hard phalanges and soft cartilage/synovial fluid between joints) can be imaged in vivo through multispectral 3D qPAT. Compared to the healthy subject (Figure 2b), an increase in hemoglobin concentration and a decrease in oxygen saturation were observed in the soft joint tissue of the finger bone and joint cavity in OA (Figure 2c). Multispectral 3D qPAT is a promising method for detecting angiogenesis and hypoxia associated with OA diseases, and a potential clinical tool for early detection of OA in finger joints.^[^ ^13^ ^]^

### 2.2 Rheumatoid Arthritis (RA)

RA is a chronic autoimmune inflammatory disease characterized by synovial arthritis. Cartilage and bone tissue are irreversibly damaged, leading to disability, inability to participate in work and social activities, increased mortality rate, and serious impact on the quality of life of patients.^[^ ^14^ ^]^ The characteristic of RA is the infiltration of T cells, B cells, and monocytes into the synovium of multiple joints. New blood vessel formation (growth of new blood vessels) is another hallmark of RA synovitis. The expansion of synovial fibroblast like cells and macrophages leads to proliferation of the synovial lining layer. This dilated synovium, commonly known as “vascular pannus”, invades the periarticular bone at the cartilage bone junction, leading to bone erosion and cartilage degradation.^[^ ^15^ ^]^

Imaging examinations play an important role in the diagnosis and treatment monitoring of rheumatoid arthritis. In clinical practice, X‐ray and CT are commonly used diagnostic tools for RA, but they can only be used in cases of bone deterioration and lack specificity for soft tissue and blood vessels.^[^ ^16^, ^17^, ^18^, ^19^ ^]^ In addition, X‐ray and CT involve radiation, which may increase the risk of cancer or teratogenicity. MRI provides higher sensitivity for identifying soft tissue and bone regeneration, as well as imaging methods for synovial lesions. MRI is more diagnostic than other methods and helps to narrow down the scope of differential diagnosis.^[^ ^20^ ^]^ But it is hindered by high cost, low temporal resolution, and narrow patient enclosed spaces, which may lead to claustrophobia.^[^ ^21^ ^]^ There is an unmet need for higher sensitivity, specificity, and non‐invasive diagnostic monitoring methods to identify synovial erosion and vascular opacity in the early stages.

**Figure 3:**
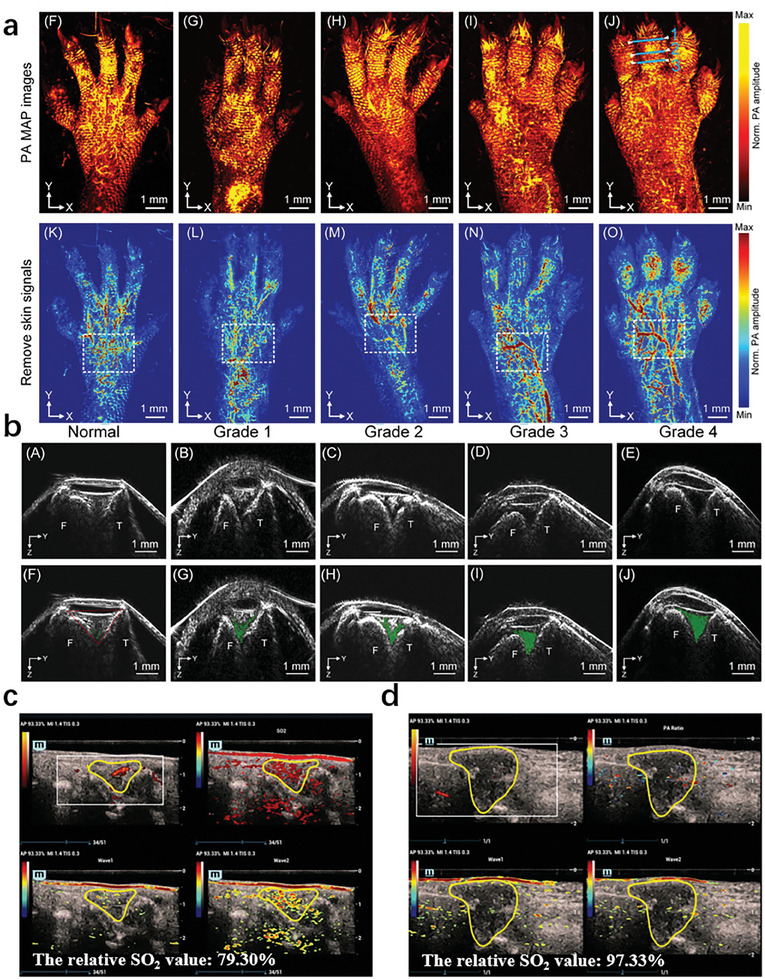
PA images of rheumatoid arthritis. a) Photoacoustic imaging of the hind paw of DBA/1 mice.^[^ ^22^ ^]^ b) The B‐ultrasound image shows synovial erosion in the joint cavity.^[^ ^22^ ^]^ c) Examples of PD 1 and PA 2.^[^ ^23^ ^]^ d) Examples of PD 2 and PA 3.^[^ ^23^ ^]^

RA is a chronic autoimmune disease characterized by neovascularization, synovial hyperplasia, and articular cartilage destruction. However, there is still a lack of characteristic early diagnosis and treatment monitoring methods. US imaging, as a mature imaging technique, has been widely used in the diagnosis, treatment, and monitoring of rheumatoid arthritis. The photoacoustic and ultrasound imaging (PA/US) systems are highly similar and can share the same ultrasound transducer to receive signals. Photoacoustic imaging provides the morphology of blood vessels within tissues, while ultrasound imaging reveals tissue structure. PA/US dual‐mode imaging can effectively avoid artifacts and provide high‐resolution and high contrast imaging for rheumatoid arthritis. By establishing a collagen induced arthritis (CIA) RA mouse model and classifying the disease status based on a subjective grading system, PA/US imaging can evaluate synovial erosion and vascular opacity in the knee joint in real‐time at high spatial resolution under different disease states. The system also quantitatively monitors the physiology and morphology of subcutaneous blood vessels in the hind paws of mice, measures the area of vascular proliferation and the intensity of photoacoustic signals, and is positively correlated with disease grading. Compared with traditional subjective scoring of arthritis severity, PA/US imaging is more sensitive, meaning that vascular signals and synovial erosion can be observed in the early stages of arthritis. In summary, their research findings indicate that PA/US dual‐mode imaging is a valuable method for evaluating RA lesions in mice. PA provides the advantage of visualizing and analyzing neovascularization in the knee joint of RA (**Figure** **3**a), while US can also quantify erosive vascular opacity in the knee joint (Figure 3b).^[^ ^22^ ^]^

Multimodal PA/US joint imaging scoring through experiments was evaluated, which is a new imaging method that can reflect the inflammatory microvascular and oxygenation levels in rheumatoid arthritis joints. The PA/US imaging system was applied to examine 7 small joints. Use a 0–3 scoring system to semi quantitatively evaluate PA and Power Doppler (PD) signals, and calculate the sum of PA and PD scores (PA sum and PD sum scores) for 7 joints (Figure 3c). Measure the relative oxygen saturation (SO_2_) value of inflammatory arthritis lesions and classify it into three PA+SO_2_ modes. Further evaluate the correlation between PA/US imaging score and disease activity score. They found that the PA score of RA patients was correlated with the standard clinical score, and the PA+SO_2_ pattern was correlated with the clinical score reflecting the severity of pain. PA may have clinical potential in evaluating RA (Figure 3d).^[^ ^23^ ^]^

### 2.3 Ankylosing Spondylitis (AS)

Axial spondyloarthritis is a chronic inflammatory disease characterized by central axial bone lesions. It is a type of spondyloarthritis that also includes psoriatic arthritis, inflammatory bowel disease‐related arthritis, and reactive arthritis. AS is a common inflammatory rheumatic disease that affects axial bones, causing characteristic inflammatory back pain that can lead to structural and functional damage and reduced quality of life. AS is characterized by inflammation and new bone formation in the axial bones.^[^ ^24^ ^]^

Arthritis and attachment point inflammation are the most common peripheral manifestations that can occur at any time during the disease process. These manifestations mainly occur in the lower limbs and are usually asymmetrical. The joints are usually swollen and painful. Inflammation at the attachment point of tendons, ligaments, or joint capsules is called attachment point inflammation. A typical location is the attachment point of the Achilles tendon or plantar fascia to the calcaneus, but inflammation may occur at any attachment point.^[^ ^25^ ^]^ Imaging examination is crucial for the correct (and early) diagnosis and differential diagnosis of axial arthritis. Because compared to the spine, this disease affects the sacroiliac joint in most patients, sacroiliac joint imaging plays a crucial role in diagnosing and further classifying axial spinal arthritis.

**Figure 4:**
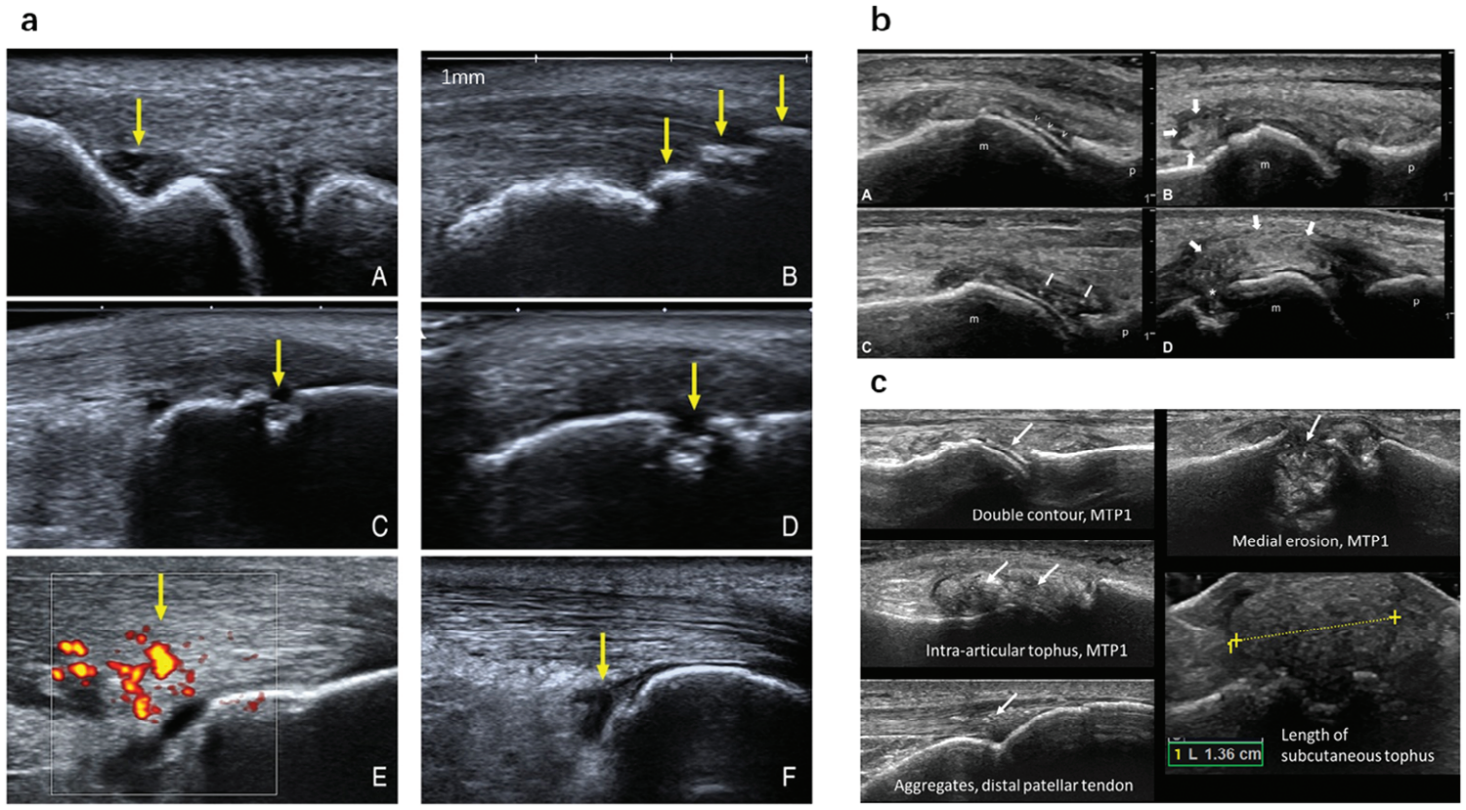
US image of AS, ReA, and gout. a) US manifestations of attachment point inflammation and ACW lesions. ACW, the anterior chest wall.^[^ ^28^ ^]^ b) US examination of four different gout patients, showing four instances of ultrasound gout lesions. m, metatarsal head; p, phalangeal base.^[^ ^40^ ^]^ c) Double contour of metatarsophalangeal joint, MTP1 joint gouty stone, accumulation of distal patellar tendon, medial erosion of the first metatarsal head, and measurement of superficial subcutaneous gouty stone length in the proximal interphalangeal joint (PIP) of the second toe.^[^ ^42^ ^]^

AS is a disease characterized by chronic inflammation, particularly in the attachment points of the spine and surrounding bones. The mechanical stiffness of affected joints reflects the progressive fusion of adjacent vertebral segments, and the reduction in range of motion of the joints leads to decreased axial movement.^[^ ^26^ ^]^ Ultrasound physicians who were unaware of the initial clinical and radiological assessments observed and measured the vascularization, resistance index (RI), and first intervertebral foramen branch of the lateral sacral artery of the sacroiliac joint (SIJ). Using relevant statistical and graphical methods, the relationship between the RI of the SIJ and the RI of the first intervertebral foramen branch of the lateral sacral artery in AS was obtained through logistic regression analysis, SPSS24.0, and MedCalc19.6.0 software, and reflected by the Co‐index receiver operating characteristic(ROC)curve or calculating the area under the ROC curve (AUC). Using MRI‐confirmed sacroiliitis as the diagnostic criterion, the consistency between the RI of the SIJ and MRI was determined using Kappa test. They found that the RI of the SIJ was a possible role in diagnosing active sacroiliitis, therefore color doppler ultrasound (CDUS) was predicted to be a promising diagnostic tool for AS compared to MRI (**Figure** **4**a).^[^ ^27^, ^28^ ^]^

### 2.4 Reactive Arthritis (ReA)

ReA was initially defined as aseptic synovitis that occurs after distal infection.^[^ ^29^ ^]^ It is usually a self‐limiting disease, but it is widely recognized that it evolves into chronic arthritis and even typical ankylosing spondylitis. ReA is divided into HLA‐B27 related and unrelated forms.^[^ ^30^ ^]^ Reactive arthritis is an inflammatory disease that manifests in young people, typically a few weeks after infection in the gastrointestinal (GI) or urogenital (GU) tract. Although it is usually self‐limiting and resolves within a few months, some people may experience persistent symptoms. ReA is classified as one of several types of spinal arthritis (SpA). Symptoms include asymmetric oligoarthritis (most common in the large joints); Extraarticular features, including attachment point inflammation, tendonitis, and uveitis; And various rashes, such as nodular erythema, hemorrhagic keratosis, and circular balanitis.^[^ ^31^ ^]^

ReA is a type of arthritis that occurs after infection in certain specific areas, such as the gut and urogenital tract. As a group of characteristics, attachment inflammation is defined as inflammation of the bone attachments of tendons, ligaments and myofascia. The figure below shows representative examples of changes in tendons and attachment points found in the study. Through the study, they found that musculoskeletal ultrasound is an inexpensive, noninvasive, and accessible imaging technique that can help identify early changes in tendons and attachment points in patients with previous infections, and provide possible diagnostic clues for reactive arthritis, thereby optimizing disease management.^[^ ^32^ ^]^

### 2.5 Gouty Arthritis

Gout is a common condition caused by the deposition of gout crystals in joints and non‐joint structures. High serum urate concentration is the most important risk factor for the occurrence of gout. Gout is characterized by intermittent severe painful arthritis (gout attacks), caused by an innate immune response to deposited monosodium urate (MSU) crystals. The central strategy for effective treatment of gout is long‐term uric acid lowering therapy to reverse hyperuricemia, leading to the dissolution of urate crystals and long‐term prevention of gout attacks.^[^ ^33^ ^]^ The deposition of MSU crystals has been detected in joints, tendons, and soft tissues using ultrasound. Microscopic examination of MSU crystals from synovial fluid or gout stones will determine the correct diagnosis.^[^ ^34^ ^]^ Due to the preferential deposition of MSU crystals in the joints and surrounding structures, especially in the first metatarsophalangeal joint, midfoot, and knee, gout typically manifests as acute and severe inflammatory arthritis in these areas.^[^ ^35^ ^]^ In some gout patients, gout stones can also form. These nodules are composed of MSU crystal deposits and chronic granulomatous inflammatory tissue, representing a high burden of MSU crystal deposition and leading to chronic joint pain and injury.^[^ ^36^ ^]^

The latest American College of Rheumatology/European League against Rheumatism (ACR/EULAR) gout classification criteria developed in 2015 use ultrasound as a method to detect MSU crystal deposits, and the results of ultrasound examinations related to gout have been described using various terms.^[^ ^37^ ^]^ However, in 2015, the Outcome Measures in Rheumatology (OMERACT) Ultrasound Group developed consensus‐based ultrasound definitions for gout lesions, and evaluated their reliability in static images and patients according to the OMERACT process.^[^ ^38^, ^39^ ^]^ These include double contour (DC) (crystal deposits on the cartilage surface), tophi (larger collections of crystals), aggregates (small crystal deposits), and erosions (cortical lesions).^[^ ^38^ ^]^ The diagnostic value of ultrasound lesions defined by OMERACT in a cohort of patients suspected of having gout was evaluated by using both MSU crystal microscopy and the ACR/EULAR 2015 classification criteria as reference standards. All four ultrasound gout lesions showed high sensitivity for the disease. Their study showed that ultrasound visualization of tophi and DC, as defined by the OMERACT Ultrasound Group, is an effective tool for the diagnosis of gout in clinical practice (Figure 4b).^[^ ^40^ ^]^

Gout attacks may lead to bone erosion. US detected erosion through the discontinuity on the bone surface (visible in two vertical planes), and in the metatarsophalangeal (MTP)1 joint, ultrasound showed a more sensitive detection of erosion in gout patients than X‐rays.^[^ ^41^ ^]^ US evaluation of MSU crystal deposition was evaluated by using a new semi quantitative scoring system. They explored the most common deposition sites through observational studies and found that during the treatment process, all different forms of ultrasound detected sediment decreased, with DC decreasing the most. MTP1 is most commonly affected, and the MTP1 erosion score is correlated with the primary lesion score at the joint level (Figure 4c).^[^ ^42^ ^]^

### 2.6 Psoriatic Arthritis (PsA)

PsA is a chronic inflammatory arthritis that can affect up to one‐third of psoriasis patients. PsA can cause attached synovitis and structural damage, leading to a significant decline in functional ability and affecting quality of life.^[^ ^43^ ^]^ PsA is a common inflammatory disease of the peripheral and axial bones. The clinical characteristics of psoriasis are diverse, including attachment point inflammation, finger (toe) inflammation, nail malnutrition, uveitis and osteoarthritis, as well as related complications such as obesity, metabolic syndrome, and cardiovascular disease. Many studies on psoriatic arthritis have focused on the skin and joints; However, in the past 10 years, significant progress has focused on the tendon end, although the tissue at which the disease begins may vary from person to person. The importance of related comorbidities and their impact on mortality has also been recognized.^[^ ^44^ ^]^

PsA is a destructive disease that can cause structural changes in joints and attachment points. It is precisely the two processes of bone erosion and attachment site formation that together lead to the characteristic structural changes observed in PsA, namely joint injury.^[^ ^45^ ^]^ The structural damage of PsA is associated with early functional decline in the disease process. PsA structural lesions include: (i) structural attachment point lesions, namely the new bone formation point near the attachment point of the joint.^[^ ^46^ ^]^ Structural attachment point lesions occur outside the joint, outside the joint capsule but still near the joint, with tendons and ligaments inserted into the periosteum. It is worth noting that in PsA patients, the relationship between new bone formation at the attachment point and functional impairment is closer compared to bone erosion.^[^ ^47^ ^]^ Inflammation at the attachment site leads to a series of events that triggers mesenchymal cell proliferation and differentiation into osteoblasts. Sometimes, this type of lesion can also establish an intermediate scaffold of hypertrophic chondrocytes, which are later reconstructed into bone. Structural attachment site lesions continue to grow in PsA and lead to the formation of bone spurs or attachment site growths, which form the characteristic clinical manifestations of PsA. It is worth noting that the attachment point vegetation is different from the bone vegetation observed in osteoarthritis, as they appear from the extraarticular attachment point, while the bone vegetation grows from within the cartilage.^[^ ^48^ ^]^ (ii) The early occurrence of bone erosion in the clinical process of PsA, as well as the delayed treatment of PsA, not only increases the burden of osteoporosis, but also increases the functional impairment of PsA patients. Erosion is usually caused by synovitis and depends on the formation of osteoclasts in the synovial microenvironment, which is promoted by macrophage colony‐stimulating factors and Receptor Activator of Nuclear Factor‐κB Ligand (RANKL) (essential factors for osteoclast growth and differentiation), as well as pro‐inflammatory cytokines such as tumor necrosis factor (TNF) and interleukin 17 (IL‐17), which further enhance osteoclast formation and inhibit bone formation.^[^ ^49^ ^]^ In addition, an increase in the concentration of pro‐inflammatory cytokines leads to an increased risk of systemic bone loss and osteoporosis.^[^ ^50^ ^]^

In PsA, ultrasound plays an increasingly important role in differential diagnosis and monitoring of treatment response. PsA is a heterogeneous disease involving different areas and specific sites. Therefore, a specialized ultrasonography comprehensive score is needed to monitor disease activity and identify structural damage progression. The anatomical structures that are ranked best are: attachment point, joint, sheath‐bearing tendon, sheath‐free tendon, and finally soft tissue and bursa. This is the first important step in evaluating the simplified US scoring content, which will include joint and extra‐articular structures, and will provide the most information in the US evaluation of PsA.^[^ ^51^ ^]^

The Classification Criteria for the Study of Psoriatic Arthritis study (CASPAR) diagnostic criteria for PsA are based on inflammatory lesions in the joints, spine, or attachment points. Traditionally, the assessment of localized inflammation in joints, attachment points, and tendons relies on physical examination. However, multiple studies have shown that ultrasound can detect subclinical inflammation as well as non‐inflammatory lesions. US examination improved the specificity of CASPAR criteria compared to physical examination. By combining nail changes on X‐ray, new bone formation, tendon sheath inflammation on ultrasound, and attachment point inflammation, the diagnosis of early PsA can be improved.^[^ ^52^ ^]^

## 3 Photoacoustic Imaging Nanoprobes for Inflammatory Orthopedic Diseases

**Figure 5:**
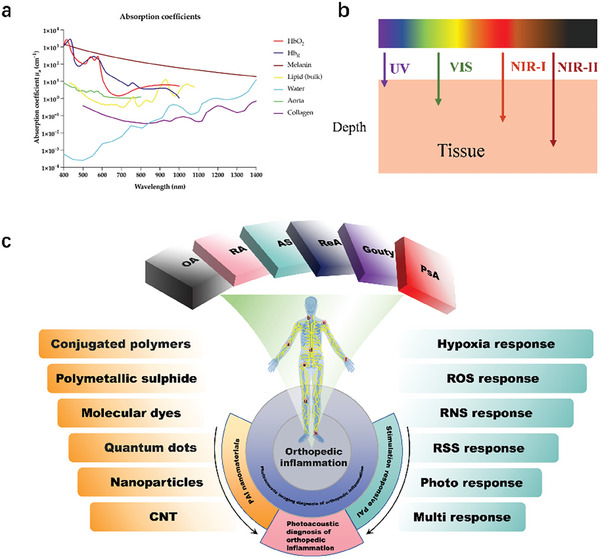
Nanomaterials for photoacoustic imaging. a) The relationship between absorption coefficient and wavelength for different photoacoustic imaging molecules and tissues.^[^ ^7^ ^]^ b) Comparison of wavelength bands from visible light to near‐infrared region and penetration range in biological tissues. c) Photoacoustic imaging for the diagnosis of orthopedic inflammation.

The contrast of PAI comes from light absorption, making it sensitive to endogenous light absorbents such as lipids, melanin, water, and hemoglobin. By utilizing these inherent absorbers as contrast agents, PAI enables imaging of blood oxygen saturation, hemoglobin concentration, melanin distribution in the skin, pigmentary skin lesions, and related microvascular networks (**Figure** **5**a). However, endogenous substances still have limitations in precise imaging and disease diagnosis. First, endogenous contrast agents exhibit individual differences due to factors such as genetic background and lifestyle, while exogenous contrast agents can be personalized according to different conditions to achieve optimal results. Second, endogenous photoacoustic contrast agents are usually present throughout the body, so their specificity is low and they may not be able to accurately distinguish different tissues or lesions. Besides, they may conflict with natural signals in the body, thereby reducing the signal‐to‐noise ratio and affecting the clarity and accuracy of imaging. Finally, due to the light absorption properties of endogenous contrast agents, the acoustic signals they generate may be mainly limited to the surface or shallow layers of biological tissues, which limits their application in deep tissue imaging. These drawbacks limit the widespread application of endogenous photoacoustic contrast agents in biomedical imaging, therefore exogenous contrast agents have been developed to improve imaging specificity, signal‐to‐noise ratio, and imaging depth. Many optical nanomaterials have been used as exogenous photoacoustic contrast agents (PACTs), such as small organic dye‐based nanoparticles (NPs), semiconductor polymer nanoparticles (SPNs), carbon‐based nanomaterials, precious metal nanomaterials, and transition metal disulfides (TMDCs). These photoacoustic imaging contrast agents can be roughly classified into organic, inorganic, and hybrid materials based on their sources. Moreover, these exogenous PACTs have the advantages of improving contrast, specificity, and penetration depth (**Table** **3**).

**Table 3:** The classification of nanomaterial‐based NIR PA contrast agents.

Category	Subcategory	Advantage	Material	Absorption peak	Reference
Organic	Assembled small molecule Conjugated polymers Organic molecular dyes	Fast excretion, low toxicity, and good biocompatibility Unique absorption and good photostability Good biocompatibility, low cytotoxicity, structural adjustability, and good biodegradability	QRu PLGA RES‐DS NPs	750nm	[53]
			M‐TDNPs	710nm	[54]
			BAF4 NPs	1003 nm	[55]
			PDPPTBZ NPs	1064 nm	[56]
			TCZ‐PNPs	1200nm	[57]
			DPPTz NPs	939 nm	[58]
			DPPQu NPs	902 nm	[58]
			Lip(PTQ) NPs	Broad peak	[59]
			Cy5.5 biotin double‐labeled VEGF antibody MBs	830nm	[60]
			Zn_4_‐H_2_Pc/DP NPs	1048nm	[61]
			P‐Pc	1064nm	[62]
Inorganic	Metal sulfides Metals	NIR‐II photothermal conversion characteristics LSPR effect, excellent light absorption and photothermal conversion ability	CuS NPs	1064nm	[63]
			MoS_2_@CS @Dex (MCD)	Broad peak	[64]
			anti‐NGF‐MoS_2_‐AuNRs	710nm	[65]
			AuPB	Broad peak	[66]
			A‐nanoceria‐ICG	Broad peak	[67]
			AuNNR@MSi‐AuNPs	PA_750_ /PA_1250_	[68]
			IL‐4@AuNCs	680nm	[69]
			AgBr@PLGA	Broad peak	[70]
	Carbon nanomaterials	Easy to manufacture and functionalize	Gd‐Fe/HCS	Broad peak	[71]
			HSC‐2	Broad peak	[72]
	Quantum dots	Good imaging effect	BQDs‐PEG	Broad peak	[73]
			N‐CQDs‐5	470nm/560nm	[74]
Hybrid		Compensate for the shortcomings of poor stability of organic materials, poor hydrophilicity of inorganic materials, and low biocompatibility.	Au@PDA‐WL NPs	720nm	[75]
			NM‐LANP @ Ru	800nm	[76]
			AuNR@PEG/PolyRu Ves	Broad peak	[77]

For biological imaging, the visible light region (400–700 nm) is the easiest to achieve, with narrow penetration (approximately 1 mm) in biological tissues. In contrast, the NIR window (700–1700 nm) has more exposure and less photon scattering, capable of penetrating several centimeters (Figure 5b). In the field of inflammatory orthopedic diseases, Photoacoustic imaging can achieve better imaging results by utilizing different materials from different levels (Figure 5c). For PAI applications, NIR ensures higher tissue penetration depth, spatial resolution and signal‐to‐noise ratio (Table 2).

### 3.1 Photoacoustic Imaging Nanoprobes for RA

**Figure 6:**
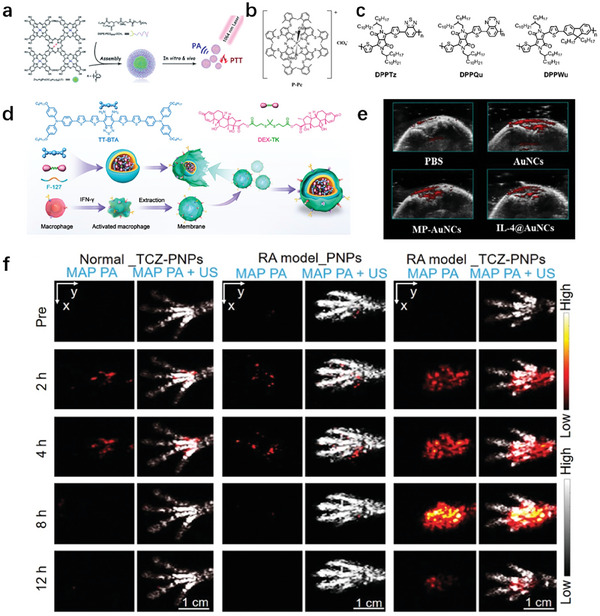
Design and development strategy of organic nanoprobes for photoacoustic imaging. a) Manufacturing instructions for Zn_4_‐H_2_Pc/DP NPs for photothermal therapy and photoacoustic imaging.^[^ ^61^ ^]^ b) phosphorus phthalocyanine (P‐Pc).^[^ ^62^ ^]^ c) Schematic diagram of the chemical structure and SPN degradation of DPPTz, DPPQu, and DPPWu, as well as normal mice and injection of DPPQu NP and DPPTz NP λ‐ Representative pie chart of the right hind foot joint in mice with carrageenan induced arthritis.^[^ ^58^ ^]^ d) Schematic diagram of therapeutic diagnostic nanomaterials for NO activatable PA imaging and synergistic treatment of rheumatoid arthritis.^[^ ^54^ ^]^ e) PA images in vitro and in vivo.^[^ ^69^ ^]^ f) MAP PA/US images.^[^ ^57^ ^]^

Nanoparticles are nanoscale carriers made of biocompatible and biodegradable polymers, which contain small molecule drugs with high targeted therapeutic potential. Generally speaking, NP is stable for an extended period of time and can load hydrophilic and hydrophobic compounds. It is possible to control the release of payloads to actively target specific cells, organs, or tissues.^[^ ^78^ ^]^ The phthalocyanine pentapeptide was designed as the PAI probe. The water‐soluble nanoparticles (Zn_4_‐H_2_Pc/DP NPs) assembled from this single‐molecule material with the help of DSPE‐PEG_2000_‐OCH_3_ exhibit characteristic absorption in the NIR‐II region at 1064 nm, with a high extinction coefficient of 52 L g^−1^ cm^−1^, a high photothermal conversion efficiency of 58.3%, and a strong photoacoustic signal (**Figure** **6**a).^[^ ^61^ ^]^ PAI probes with absorbance exceeding 1000 nm can be prepared using phthalocyanine phosphorus (P‐Pc). By utilizing the widely available 1064 nm pulse laser emission output, photoacoustic computed tomography (PACT) can image P‐Pc of 11.6 cm chicken breast. When the contrast agent is placed under the arm of a healthy adult, PACT transducer at the top of the arm can easily detect P‐Pc across the entire 5 cm limb. Therefore, the method of using contrast agents with extreme absorption at 1064 nm can easily achieve high‐quality optical imaging of the human body at special depths in vitro and in vivo (Figure 6b).^[^ ^62^ ^]^

Semiconductor polymers can be used as contrast agents for PAI due to their high extinction coefficient and long absorption band. Semiconductor polymers (SP) are linear long chains formed by the polymerization of conjugated electron donating units and electron acceptor units.^[^ ^79^ ^]^ It is precisely this long linear conjugated structure that makes it easier for SP to exhibit redshift absorption wavelength and high molar extinction coefficient. Biodegradable semiconductor polymers with unique optical properties can be designed for PAI. The optical properties of semiconductor polymers are regulated by designing their molecular structures. Polymers with donor‐π‐acceptor structures can improve their optical properties by adjusting the donor or acceptor units. Three types of diketone pyrrole derivative polymers (DPPTz, DPPQu, and DPPWu) were synthesized by adjusting the electron donating and acceptor units, and nanoparticles were prepared by nanoprecipitation method. Among them, DPPTz and DPPQu have conjugated D‐A_1_‐D‐A_2_ structures, while DPPWu has conjugated donor‐acceptor (D‐A) structures. DPPTz NP and DPPQu NP have a wide absorption range of 600–1000 nm in the NIR window. DPPTz NP can exhibit stronger PA signals in the NIR window. Therefore, DPPQu and DPPTz nanoparticles are biocompatible and highly safe photoacoustic imaging contrast agents for RA (Figure 6c).^[^ ^58^ ^]^

Many near‐infrared absorbers based on organic materials are widely used in research due to their excellent biodegradability, biocompatibility, and structural adjustability. D‐A organic molecule with nitric oxide (NO) tunable intramolecular charge transfer and conformational changes can be designed to combine PAI activated by the RA microenvironment with multi‐target synergistic therapy for RA. The nanoprobes can detect endogenous NO in RA lesions in real‐time and sensitively, and distinguish the severity of RA (Figure 6d).^[^ ^54^ ^]^

Metal nanomaterials have excellent light absorption and photothermal conversion capabilities due to the localized surface plasmon resonance (LSPR) effect, which can be used for PAI. When exposed to light of an appropriate wavelength, conductive electrons on the surface of nanoparticles oscillate with each other at the resonance frequency of the cationic lattice. The oscillation energy is mostly converted into the thermal energy detected by photoacoustic imaging, and its molar absorption coefficient is several orders of magnitude higher than that of small molecule dyes. It is the contrast agent with the highest molar absorption coefficient in the NIR range and has high photoacoustic conversion efficiency. Precious metal nanoparticles mainly include gold and silver nanoparticles, among which gold nanoparticles (GNP) are widely used in photoacoustic imaging. The PAI nanoprobes was synthesized by grafting Polyethylene glycol (PEG) functionalized folate (FA) and conjugating interleukin 4 (IL‐4) onto gold nanocages (AuNCs). Among them, AuNCs conjugates can serve as contrast agents for PA phantom and CT imaging. PAI was performed using IL‐4@AuNC of different concentrations at a laser wavelength of 680nm. As the concentration increased, a significantly enhanced PA signal was recorded. The nanoplatform demonstrates promising potential as a targeted therapy for RA. Accordingly, PAI using this novel AuNCs conjugate could improve the detection and diagnosis of RA (Figure 6e).^[^ ^69^ ^]^

The anti‐rheumatic targeted drug tocilizumab (TCZ) with polymer nanoparticles (PNPs) was conjugated for PA imaging guided treatment of RA. TCZ‐PNPs have good photostability and physiological stability, high absorption coefficient, good biocompatibility, and ideal PA properties, which can be used for PA imaging of deep tissues in vivo. The high inflammation specificity of TCZ‐PNPs has great advantages in the differential diagnosis of early RA and other inflammatory diseases. The NIR‐II PAI of RA joint tissue using TCZ‐PNPs showed clear visualization of swelling and cartilage tissue, indicating its diagnostic potential for non‐invasive monitoring of RA disease progression (Figure 6f).^[^ ^57^ ^]^

### 3.2 Photoacoustic Imaging Nanoprobes for OA

**Figure 7:**
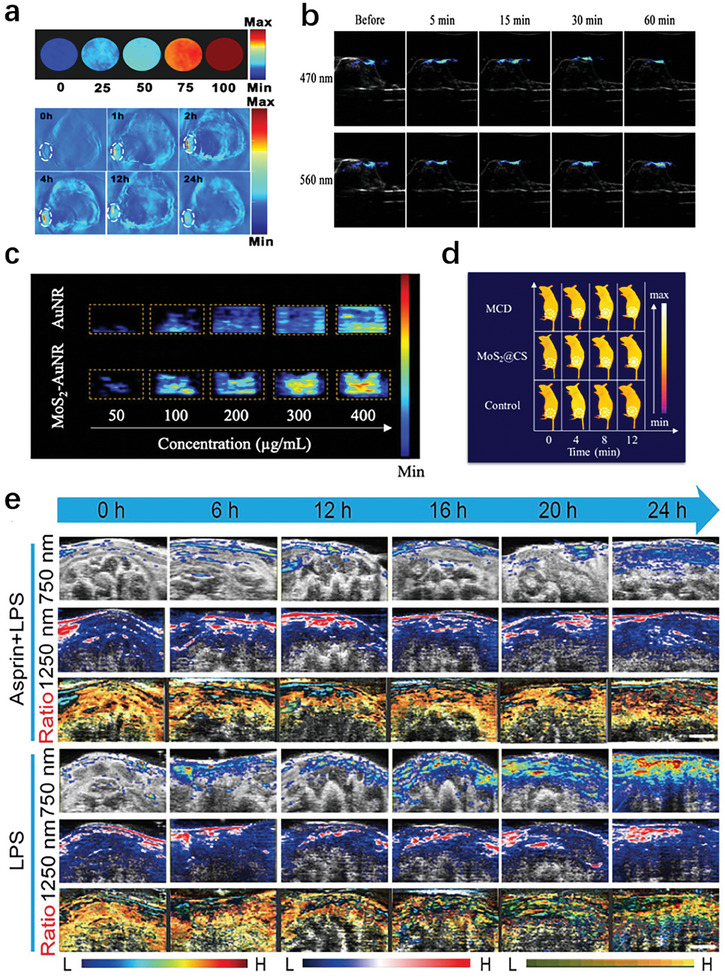
Design and development strategy of inorganic nanoprobes for photoacoustic imaging. a) The photoacoustic imaging characteristics of N‐CQD.^[^ ^73^ ^]^ b) Photoacoustic imaging of N‐CQD reserve solution at excitation wavelengths of 470 and 560 nm, respectively.^[^ ^74^ ^]^ c) Normalized PA amplitudes of AuNR and MoS_2_‐AuNR at different wavelengths.^[^ ^65^ ^]^ d) Study on the photothermal effect and retention time of MCD in mouse joints.^[^ ^64^ ^]^ e) Real time imaging of inflammation and tracking of in vivo anti‐inflammatory processes.^[^ ^68^ ^]^

Carbon nanomaterials have been widely used as biological imaging probes due to several expected properties, such as wavelength dependent luminescence emission, low light bleaching, low toxicity, suitable in vivo clearance, high stability, and solubility.^[^ ^80^ ^]^ The carbon quantum dots (CQDs) have unique structures, high specific surface area, unique optoelectronic properties, and good biosafety. Various new quantum dots, such as graphene oxide quantum dots, black phosphorus quantum dots, antimonene quantum dots have been successfully prepared and used in the biomedical field.^[^ ^81^, ^82^, ^83^, ^84^ ^]^ Boron quantum dots (BQDs) were designed to investigate their potential as PAI contrast agents. The as‐prepared PEG modified BQDs (BQDs‐PEG) can be seen that the intensity of the photoacoustic signal increases with the increase of BQDs‐PEG concentration, indicating the good PAI effect of BQDs‐PEG in vitro (**Figure** **7**a).^[^ ^73^ ^]^ CQDs are indispensable carbon nanomaterials widely used in biological PAI due to their tunable fluorescence, abundant surface charge and chemical groups, low cost, and good biocompatibility. A series of new nitrogen‐doped carbon quantum dots (N‐CQDs) through molecular fusion strategies was developed, and N‐CQDs exhibits fast PAI speed, rapid metabolic processes, and large enhanced permeability and retention (EPR) effects (Figure 7b).^[^ ^74^ ^]^

Among the numerous PAI nanomaterials, molybdenum disulfide nanosheets (MoS_2_) have attracted much attention due to their simple preparation, low cost, and excellent PAI properties. MoS_2_ nanosheets coated with gold nanorods (AuNR) can be developed for pain PAI. The MoS_2_ coating significantly improved the photoacoustic properties of AuNR. This molecular therapy diagnostic method can specifically locate the source of OA pain and effectively block the transmission of peripheral pain (Figure 7c).^[^ ^65^ ^]^ The PAI ability of MoS_2_ nanosheets can be used to detect the metabolism of MoS_2_@CS@Dex (MCD) in the joint cavity, avoid rapid clearance of the lymphatic system in the joint cavity, and prolong the drug's residence time in the joint cavity (Figure 7d).^[^ ^64^ ^]^

Metal nanomaterials can be effectively used as contrast agents for PAI. PA diagnostic nanoprobes based on cerium dioxide and nuclear satellite gold nanostructure were applied for the prevention and treatment of OA. The uniqueness of nano therapeutic agents lies in their antioxidant and enzymatic properties, which are due to the formation of new enzyme structures by albumin and nano ceria. Importantly, nanoparticles are non‐invasive and traceable for evaluating treatment delivery and biological distribution through optical/photoacoustic imaging (Figure 7e).^[^ ^67^, ^68^ ^]^

**Figure 8:**
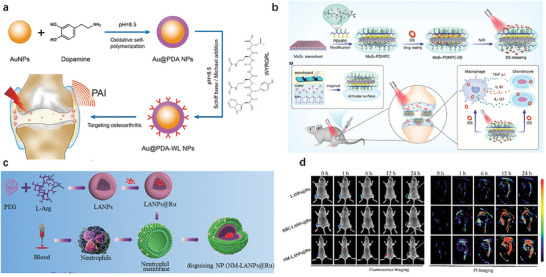
Design and development strategy of hybrid nanoprobes for photoacoustic imaging. a) Synthesis Au@PDA‐WL Schematic diagram of NP and its application in osteoarthritis.^[^ ^75^ ^]^ b) Schematic diagram of molybdenum disulfide dosing system.^[^ ^85^ ^]^ c) A schematic diagram of biomimetic nanoparticles based on hydrophilic membranes for targeted recognition of inflammatory osteoarthritis, with dual‐mode imaging diagnosis and NO gas therapy, as well as a scheme for synthesizing NM‐LANP@Ru.^[^ ^76^ ^]^ d) In vivo fluorescence imaging of KM mouse osteoarthritis models injected with different groups at different times and in vivo PA imaging of KM mouse osteoarthritis models injected with different groups at different times.^[^ ^76^ ^]^

Organic materials have the disadvantage of poor stability, while inorganic materials have poor hydrophilicity and low biocompatibility. Therefore, combining organic materials with inorganic materials can compensate for their shortcomings. The PAI probe based on organic‐inorganic hybrid nanoparticles was used to monitor the bone joint for early degeneration of articular cartilage. The injected plasmonic nano‐probe (Au@PDA‐WL NPs) can target collagen II on the surface of articular cartilage matrix, and AuNPs aggregate, resulting in a significantly increased absorption cross‐section of the nanoprobes in the near‐infrared region, demonstrating outstanding PAI ability. In addition, the catechol structure in the polydopamine (PDA) shell can clear ROS and effectively delay the development of OA (**Figure** **8**a).^[^ ^75^ ^]^

The concept of solid‐liquid composite lubrication was applied to the treatment of OA and a photoresponsive biomimetic “nano ski” (MoS_2_‐PDMPC‐DS) was successfully synthesized, which can continuously exert dual functions of anti‐inflammatory and lubrication. They modified organic biomimetic polymers with lubricating properties onto inorganic MoS_2_ nanosheets and formed a hydration layer on the surface, improving the stability of the nanosheets and endowing them with dual properties of solid‐phase and liquid‐phase lubrication. Among them, MoS_2_ has a 2D layered structure and photothermal properties, which can be used as a solid lubricant and drug carrier (Figure 8b).^[^ ^85^ ^]^ The biomimetic nanoprobe (NM‐LANP@Ru) based on lipophilic membranes was developed for inflammation OA targeted dual‐mode imaging and NO therapy (Figure 8c). In vivo studies have shown that NM‐LANP@Ru therapy effectively targets inflamed OA through dual‐mode fluorescence and photoacoustic (FL/PA) imaging with high spatial resolution and deep penetration, ultimately exhibiting excellent anti‐inflammatory activity. This nano platform may provide new treatment methods for the early diagnosis and treatment of osteoarthritis (Figure 8d).^[^ ^76^ ^]^

## 4 Changes in Orthopedic Inflammatory Information Captured by PAI

Due to the characteristic absorption spectra of different tissue components and their arrangement and distribution, PAI can be applied to observe structural changes related to arthritis, based on their wavelength dependence. Early diagnosis of arthritis helps patients alleviate pain in a timely manner and improve treatment effectiveness. However, early arthritis may exhibit minimal or very slight structural changes, which may not be easily detected or detected by PA. In this case, capturing changes in the microenvironmental parameters of OA joints, such as metabolism, blood information, and light absorption, is of crucial significance.^[^ ^86^ ^]^

### 4.1 Cartilage Changes

**Figure 9:**
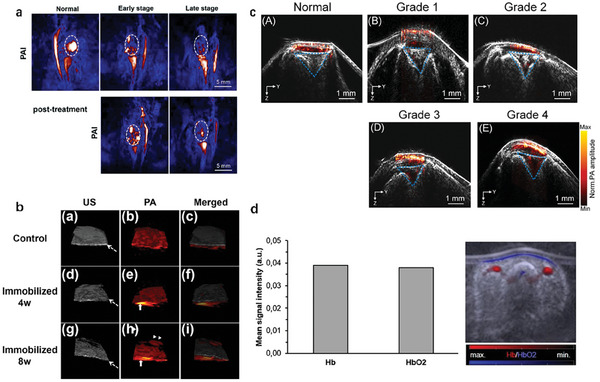
Changes in orthopedic inflammatory information captured by PAI. a) PA images of cartilage degeneration in early and late OA.^[^ ^87^ ^]^ b) Comparison of US, PAI, and PA signal intensity of subchondral bone.^[^ ^88^ ^]^ c) Photoacoustic ultrasound dual‐mode examination of the knee joint in RA DBA/1 mice.^[^ ^22^ ^]^ d) The situation of a patient.^[^ ^95^ ^]^

The PAI reagent based on endogenous melanin nanoparticles (MNPs) was developed to accurately observe cartilage degeneration in vivo. The MNP encapsulated by poly‐_L_‐lysine (PLL) is easy to manufacture, biocompatible, and has good PA strength. Through its strong electrostatic interaction with anionic glycosaminoglycans (GAGs) in cartilage, it is used for precise PAI of cartilage degeneration. Interestingly, PAI not only provided sufficient anatomical details of the knee joint, but also showed significantly higher PA intensity in the cartilage area of the normal joint compared to the OA joint. This difference is attributed to the reduced GAG levels in early OA joints, further confirmed by safranin O staining of articular cartilage. Compared with X‐ray and MRI, the use of poly‐_L_‐lysine‐enveloped MNPs (PLL‐MNPs) to detect biochemical changes in cartilage degeneration indicates true early diagnosis of cartilage degeneration. The in vivo experimental results indicate that PLL‐MNPs enhance cartilage degeneration and provides accurate information on cartilage degeneration, as well as effectively distinguishing early and late cartilage degeneration. In addition, visualizing the changes in GAG content in articular cartilage can also allow for in vivo monitoring of the therapeutic effect of osteoarthritis (**Figure** **9**a).^[^ ^87^ ^]^

PAI can also be applied to evaluate articular cartilage and subchondral bone. With the extension of braking time, the surface of the joint cartilage becomes irregular, and no subchondral bone is observed in US imaging (the white dashed arrow indicates subchondral bone). On the contrary, the PAI mode shows a significant increase in signal in the subchondral bone and shows prolonged immobilization. The quantitative PA changes can be observed that there is a significant difference in signal intensity from the subchondral bone before and after braking. Therefore, the 3D imaging of PA signals helps to diagnose abnormalities in subchondral bone and evaluate OA joints (Figure 9b).^[^ ^88^ ^]^

### 4.2 Vascular and Hypoxic States

The hypoxia detected by PAI in thickened synovium is associated with less vascularization and higher disease activity in patients with RA. Organizational hypoxia, also known as tissue level hypoxia, is a hallmark of inflammatory diseases including RA.^[^ ^89^, ^90^ ^]^ Multimodal PA/US imaging was conducted to evaluate the tissue oxygenation status of thickened synovium in patients with RA. In this prospective study, 118 participants with RA and 15 control participants were classified as hyperoxia, moderate oxygenation, or hypoxia based on synovial oxygen saturation values measured by PAI. Their results indicate that thickened synovium with higher levels of oxygenation has richer Doppler ultrasound display of blood vessels than hypoxia and intermediate synovium. In summary, PAI detection of hypoxia in thickened synovium is associated with less local vascularization and higher disease activity in RA.^[^ ^91^ ^]^

Previous studies have shown that PAI on the knee joints of normal mice and RA mouse models with varying degrees of RA. The research results indicate that PA/US dual‐mode imaging is a valuable method for evaluating RA lesions in mice. PAI provides the advantage of visualizing and analyzing neovascularization in the knee joint of RA, while US can also quantify erosive vascular opacity in the knee joint. In the photoacoustic images of normal mice, a very small amount of punctate photoacoustic signals can be seen in the tibiofemoral tendon‐tibiafemur (TTF) region. In RA mice, two to three large dot shaped photoacoustic signal regions attached to the surface of the femoral and tibial joints can be seen in the TTF region of mice with a disease status score of 1. In mice rated 2, more punctate lamellar photoacoustic signal regions were observed in the TTF region attached to the femoral and tibial joint surfaces. At a score of 3, linear regions of photoacoustic signals along the surface of the femoral and tibial joints can be observed in the TTF region. In mice rated 4, patchy areas of photoacoustic signals extending towards the center of the joint cavity can be observed in the TTF region. Compared with normal mice, the neovascularization of synovium, meniscus, and subchondral bone in the RA mouse model gradually increased. Besides, the photoacoustic signal area of RA mice increased with the increase of disease score. The intensity of the photoacoustic signal reflects the density, diameter, and flow rate of blood vessels. The area and intensity of photoacoustic signals are positively correlated with disease scores (Figure 9c).^[^ ^22^ ^]^

### 4.3 Hydrogen Peroxide

Inflammation is a complex biological response of body tissues to harmful stimuli such as pathogens, damaged cells, or stimuli, and produces a large amount of ROS, including superoxide anions, hydroxyl radicals, and H_2_O_2_.^[^ ^92^ ^]^ Hydrogen peroxide is closely related to many important physiological and pathological events. Liposome PA nanoprobes were designed for in vivo H_2_O_2_ responsive colorimetric determination. PA imaging of H_2_O_2_ related inflammatory processes induced by LPS or bacteria in mice was achieved. They prepared a liposome nanocarrier (Lipo@HRP&ABTS) that simultaneously loaded HRP and its substrate ABTS for in vivo PA imaging detection of H_2_O_2_. Through validation in inflammatory disease models, it has been demonstrated that Lipo@HRP&ABTS nanoprobes have significant advantages in H_2_O_2_ photoacoustic imaging detection.^[^ ^93^ ^]^

### 4.4 Others

Multispectral photoacoustic tomography (MSOT) on 22 psoriasis patients and 19 healthy volunteers was performed to evaluate the presence of arthritis by quantifying the photoacoustic signal intensity of endogenous chromophore oxygenated hemoglobin and deoxyhemoglobin. There is one middle‐aged psoriasis patient who has not started systemic treatment and one middle‐aged female psoriasis patient who shows signs of inadequate treatment response. The quantitative analysis of their average signal intensity showed an increase in the levels of two types of hemoglobin, but no signs of arthritis were found in these two patients on X‐ray examination. MSOT follow‐up imaging was performed in two patients three months after induction therapy with either secukinumab or methotrexate. In these two patients, a decrease in signal intensity was detected. The decrease in signal intensity corresponds to subjective pain relief (Figure 9d).^[^ ^94^, ^95^ ^]^

## 5 PAI Based on Pathological Microenvironment Regulation of Inflammatory Orthopedic Diseases

**Figure 10:**
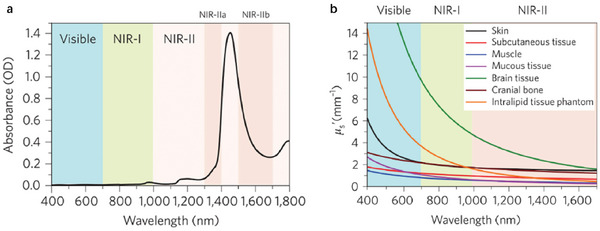
Bioimaging window.^[^ ^96^ ^]^ a) The absorption spectrum of water passing through a 1 mm long path. b) The scattering coefficients of different biological tissues and intra class scattering tissue models decrease with wavelength variation in the range of 400–1700 nm.

For intracorporeal imaging, deeper penetration, less background signal, and higher spatial resolution are necessary. In fact, tissue absorption and scattering can be minimized in two well‐defined spectral ranges—the “biological window”, the so‐called first near‐infrared (NIR‐I, 700–900 nm) and second near‐infrared (NIR‐II, 1000–1700 nm) windows (**Figure** **10**).^[^ ^96^ ^]^

Biological tissues have varying degrees of light absorption and scattering capabilities. Once incident light is irradiated, the absorption and scattering capabilities of biological tissues make it difficult for the incident light to reach the depths of most tissues. After understanding, compared with visible light and NIR‐I light, the scattering and absorption of NIR‐II incident light in biological tissues are significantly reduced. In other words, the penetration depth of NIR‐II light (5‐20 mm) is significantly deeper than visible light (1.1 mm) and NIR‐I light (3.5 mm).^[^ ^97^ ^]^ Overall, the NIR‐II window provides better light penetration, lower background signal, and higher sensitivity. What's more, biological tissue is more resilient to longer wavelengths of light, and the NIR‐II window has a larger maximum allowed exposure (MPE). Therefore, NIR‐II light is more suitable for deep tissue imaging.

Those reagents that can only produce a constant photoacoustic signal during excitation are called “normally open” contrast agents. These “always on” reagents produce stable photoacoustic signals, regardless of whether they are bound to or near the target of interest (such as the disease site). Therefore, they themselves may cause strong background noise and cannot accurately reflect biological activity at the molecular level. On the other hand, activatable contrast agents (also known as intelligent contrast agents) can produce changes in physical properties (such as absorption wavelength, absorption intensity, non‐radiative relaxation conversion, or fluorescence quantum yield), and subsequently cause changes in photoacoustic signals. Unlike “always on” contrast agents, activatable contrast agents can only be activated by specific biological stimuli (such as changes in enzyme, ion, reactive oxygen/nitrogen species, pH value, etc.) and have the advantages of low background noise and real‐time tracking of biological dynamics.^[^ ^98^ ^]^ In addition, bone diseases often have multiple physicochemical/biochemical characteristics, and the development of dual/multiple stimulus response systems can provide synergistic nonlinear responses to enhance therapeutic efficacy.^[^ ^99^ ^]^ The significant biochemical characteristics of the microenvironment have been used to develop stimulus response systems for spatiotemporal drug delivery, and even directly alter the pathological microenvironment for the treatment of bone diseases. The combination of drug release and microenvironmental characteristics helps to better capture the pathological stages of bone diseases and maximize the therapeutic potential of drug molecules.^[^ ^100^ ^]^

### 5.1 Hypoxia and ROS Regulation

**Figure 11:**
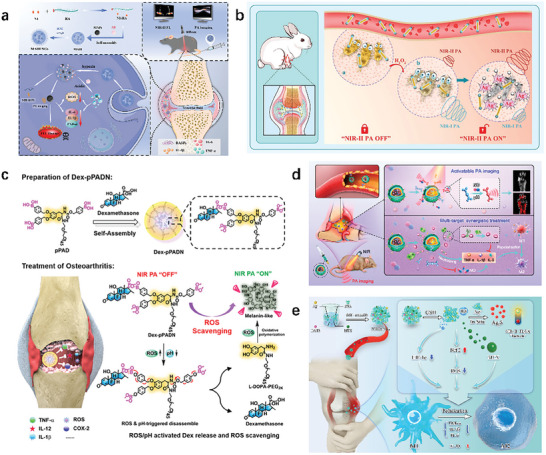
Application of PAI based on pathological microenvironment regulation. a) Schematic diagram of the synthesis of MAHI NG and its application in comprehensive treatment of RA.^[^ ^101^ ^]^ b) The ratio PA imaging (PA 1260/PA 700) at 1260 and 700 nm accurately quantified H_2_O_2_ in bacterial infections and abdominal inflammation (even osteoarthritis). The NIR‐II PA image activated by H_2_O_2_ can further accurately distinguish between inflammatory regions and normal tissues.^[^ ^102^ ^]^ c) Dex‐pPADN for the treatment of osteoarthritis.^[^ ^103^ ^]^ d) Schematic diagram of therapeutic diagnostic nanomaterials for NO activatable PA imaging and synergistic treatment of rheumatoid arthritis.^[^ ^54^ ^]^ e) Schematic diagram of BDMA NG preparation and its advanced strategy for activating NIR‐II FL/PA imaging guided synergistic therapy through M1 to M2 repolarization.^[^ ^107^ ^]^

ROS has been proven to play a role in various physiological processes and diseases, including cell apoptosis and inflammation. Therefore, ROS detection is crucial in oxidative stress‐related diseases. A low oxygen responsive nanoformulation (MAHI NGs) based on MNP/AB/2‐nitroimidazole‐hyaluronic‐acid (NI‐HA) was developed, whose surface was modified with indocyanine green (ICG) for achieving NIR‐II FL/PA imaging guided multiple therapies for RA. Due to its good stability and superior biocompatibility, MAHI NGs achieved effective accumulation in the RA region of CIA mice through leakage of the vascular system and subsequent inflammatory cell‐mediated isolation (ELVIS) effect (**Figure** **11**a).^[^ ^101^ ^]^ The excessive production of reactive oxygen species such as H_2_O_2_ is the pathological basis for chronic inflammatory diseases. H_2_O_2_ responsive therapeutic diagnostic nanoprobe was prepared by using Ag shell coated palladium tipped gold nanorods (Au‐Pd@AgNR). The ratio PAI at 1260 and 700 nm (PA 1260/PA 700) was used to quantify H_2_O_2_ in abdominal inflammation mouse models and rabbit osteoarthritis models (Figure 11b).^[^ ^102^ ^]^ A nanoparticles (Dex‐pPADN) was developed to serve as an effective PA contrast agent for non‐invasive tracking and monitoring of OA treatment through ROS triggered structural changes. pPADN provides a scaffold reversibly coupled with glucocorticoids to promote ROS‐ and pH‐responsive self‐assemblies of glucocorticoids based on 1,3‐diol/phenylboronic acid chemistry and hydrophobic interaction (Figure 11c).^[^ ^103^ ^]^

### 5.2 Reactive Nitrogen Species (RNS) and Reactive Sulfur Species (RSS) Regulation

Given the complex pathogenesis of RA, it is now recognized that targeting multiple pathological pathways is necessary to delay the uncontrolled progression of RA.^[^ ^104^ ^]^ More and more evidence suggests that in the context of RA, toxic oxygen free radicals such as NO are locally overproduced.^[^ ^105^ ^]^ The sustained presence of NO can induce chondrocyte apoptosis and damage cartilage, as well as recruit immune cells to enhance inflammation. The increase in NO levels in joints provides a promising molecular target for the diagnosis and treatment of RA.^[^ ^106^ ^]^ The probes with NO activatable signals and amplified PA conversion were developed by assembling them with ROS responsive dexamethasone (Dex) prodrugs. The combination of probe mediated NO clearance and on‐demand Dex prodrug activation can greatly inhibit pro‐inflammatory factors, significantly reducing RA symptoms in CIA models. This carefully designed nanomedicine cleverly combines RA specific PA molecular imaging with synergistic multi‐target therapy, providing great hope for precise intervention in RA related diseases (Figure 11d).^[^ ^54^ ^]^ The therapeutic operation of gas transmitter hydrogen sulfide (H_2_S) has shown promising potential as an innovative strategy for RA, but is limited by non‐targeting, low solubility, and systemic toxicity. Multifunctional glutathione (GSH)‐responsive H_2_S nanogenerator (BDMA NG) based on stimulus responsive nanotechnology was developed, achieving precise NIR‐II FL/ PA bimodal imaging guided gas/chemotherapy synergistic therapy that can be activated. The non‐adsorption of H_2_S and the in‐situ mineralization process of Ag^+^ ions lead to the rapid formation of Ag_2_S to “open” the NIR‐II FL/PA signal (Figure 11e).^[^ ^107^ ^]^

### 5.3 Photo‐Regulation

Photosensitive liposome can respond to NIR light both in vitro and in vivo, and regulate the radiation behavior of NIR light. Molybdenum (Mo) based polyoxyethylene oxide (POM) clusters, often described as nanoclusters of transition metals and oxygen atoms, have been shown to be promising candidates for ROS related diseases.^[^ ^108^, ^109^ ^]^ In addition, some studies have demonstrated that POM has excellent NIR response characteristics, making it a promising candidate for the treatment of various diseases.^[^ ^110^ ^]^ Interestingly, NIR also synergistically increases local temperature to treat arthritis.^[^ ^111^ ^]^

**Figure 12:**
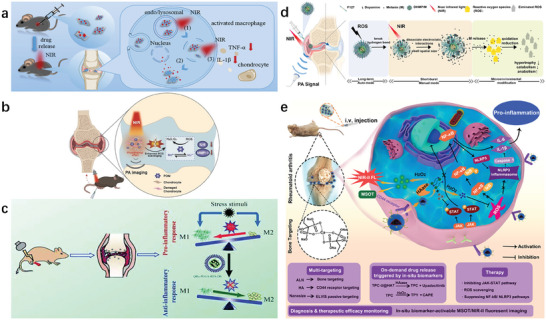
Application of PAI based on pathological microenvironment regulation. a) The mechanism of nanosystem responding to NIR light for the treatment of osteoarthritis in vivo through intra‐articular injection.^[^ ^64^ ^]^ b) Schematic diagram of OA treatment through NIR responsive POM nanoclusters.^[^ ^112^ ^]^ c)Research on QRu‐PLGA‐RES‐DS NPs for the treatment of RA.^[^ ^53^ ^]^ d) A schematic diagram of DHMP/M as a ROS/NIR dual stimulus responsive drug delivery system for the long‐term and short‐term burst release of knee osteoarthritis guided by photoacoustic imaging.^[^ ^118^ ^]^ e) Schematic diagram of multi‐target, dual biomarker response detection/imaging and treatment for rheumatoid arthritis.^[^ ^123^ ^]^

During reduction or oxidation, Mo ions in POM exhibit valence states that are prone to change between Mo^5+^and Mo^6+^. Here, the intra‐articular drug delivery nanosystem (MCD) was synthesized by using chitosan (CS) modified molybdenum disulfide (MoS_2_) nanosheets as near‐infrared (NIR) photo‐responsive carriers, loaded with anti‐inflammatory drug dexamethasone (Dex). The photoacoustic imaging capability of MoS_2_ nanosheets can be used to detect the metabolism of MCD in the joint cavity. Because MCD was response to NIR light both in vitro and in vivo, Dex release could be triggered through photothermal conversion (**Figure** **12**a).^[^ ^64^ ^]^ The NIR responsive molybdenum‐based polyoxometalate (POM) exhibits significant ROS clearance through temperature increase. The results further demonstrate the superior therapeutic effect of NIR in response to POM on OA, which reduces inflammatory cytokines and metabolic proteases in articular cartilage. Due to its strong absorption of near‐infrared, POM has been proven to be an excellent PA tracer in both in vivo and in vitro experiments (Figure 12b).^[^ ^112^ ^]^

The photothermal effect as a drug controlled release has received significant attention. The photothermal response system can only release drugs under the photothermal effect when stimulated by external light sources. Compared to endogenous stimuli, exogenous light stimulation is more stable and has advantages in precise remote control of drug release.^[^ ^113^ ^]^ A core‐shell PAI nanoprobe (QRu‐PLGA‐ES‐DS NPs) was developed to be used as the core and the target molecule sulfate dextran (DS) modified by thermosensitive molecule poly (lactic acid co glycolic acid) (PLGA) is used as the shell. The PAI nanoprobe can provide imaging guidance for the distribution and analysis of medicines in inflammatory tissues (Figure 12c).^[^ ^53^ ^]^

### 5.4 Multi‐Regulation

Multi stimulus response can greatly improve the efficiency of drug delivery to joint disease sites. Single stimulus triggering may lead to non‐specific drug leakage. On the contrary, multi stimulus responses can provide multiple insurances for on‐demand drug release at the lesion site. Once drug containing nanoparticles reach the lesion of joint disease, on‐demand release of active drugs triggered by in situ pathological stimulation will be an ideal choice for precise treatment of arthritis.

Oxidative stress is involved in the occurrence and development of OA microenvironment, leading to cartilage degradation and destruction.^[^ ^114^ ^]^ Pro‐inflammatory cytokines such as interleukin‐1β (IL‐1β) are produced in arthritis joints, leading to upregulation of ROS, followed by an increase in catabolic enzymes, disruption of extracellular matrix (ECM) synthesis homeostasis, and exacerbation of joint inflammation and chondrocyte death.^[^ ^115^ ^]^ At present, many antioxidant supplements and free radical scavengers have become promising and powerful treatments for osteoarthritis, which work by reducing oxidative stress and alleviating inflammation in the pathogenesis.^[^ ^116^, ^117^ ^]^ An intelligent antioxidant for PA imaging was designed by constructing a dual responsive mixed micelle with free radical scavenger melanin at the core of the micelle and the outer shell of polydopamine (named DHMP/M) to guided knee osteoarthritis treatment (Figure 12d).^[^ ^118^ ^]^

At present, several polymers responsive to hyaluronidase (HAase) have been studied as drug delivery systems to release therapeutic drugs for RA treatment when triggered by HAase in inflamed joints.^[^ ^119^ ^]^ The RA microenvironment contains high levels of ROS, with H_2_O_2_ being the most abundant and stable ROS member, and overexpressed enzymes (such as HAase).^[^ ^120^, ^121^, ^122^ ^]^ Therefore, using in situ H_2_O_2_ and HAase as dual pathological stimuli is an ideal method for accurately triggering on‐demand drug release at the site of RA lesions. A nanosystem (TPC‐U@HAT) can be used for imaging and treatment of RA, which can response to dual pathological biomarkers of RA, hyaluronidase, and ROS. The pathological biomarkers present at the RA site trigger the activation of an aggregation‐induced emission (AIE)‐active NIR‐II chromophore TPY, which is used for MSOT and NIR‐II fluorescence imaging to achieve disease lesion detection and therapeutic efficacy monitoring (Figure 12e).^[^ ^123^ ^]^

## 6 Conclusion and Outlook

Many traditional medical imaging methods, including X‐ray, MRI, and ultrasound, are often used in medical settings to screen for diseases, diagnose patients, and track the effectiveness of treatment. X‐rays are faster than MRI and mainly provide information about bone structure; The soft tissue resolution of MRI is higher than that of X‐ray, which can provide richer and more detailed soft tissue information, such as cartilage and ligaments. US has been widely used in clinical internal tissue imaging for further diagnosis and treatment of diseases. The safety and non‐invasive nature of US have been widely applied in on‐demand remote drug delivery to trigger deep tissue penetration, and have been recognized. However, low spatial resolution US imaging hinders precise biomedical applications. Photoacoustic imaging is a biological tissue imaging technique that utilizes ultrasound and optical vibration enhancement to achieve high resolution and sensitivity. PAI provides a multi‐scale perspective on the inflammatory process, monitoring the structural and functional aspects of different anatomical positions, resolutions, and imaging depths. In addition to visualizing highly absorbable hemoglobin and vascular structure, future methods will also focus on novel endogenous and exogenous PA compounds. PAI is sensitive enough to detect even minor inflammatory changes in tissues, enabling early and even preventive treatment strategies.

The photoacoustic method has shown great potential in vascular imaging of small animals (whole body) and humans (surface). When conducting animal experiments, photoacoustic imaging can achieve the following effects and have good display effects on experimental drugs or materials: I. Monitor the release and distribution of drugs or nanomaterials in vivo. The high‐resolution imaging of photoacoustic imaging can clearly observe the release process of targeted drugs or nanomaterials after implantation; II. Evaluate the targeted aggregation ability of drugs or materials. By comparing the signal intensity of drugs or nanomaterials in different parts of the body, their affinity with the target tissue can be intuitively reflected; III. Observe the biosafety of drugs or materials. Through imaging, it is possible to directly observe whether drugs exhibit non‐specific aggregation of normal tissues; IV. Dynamic monitoring of treatment response process and application of multimodal image fusion. Photoacoustic imaging can be combined with other imaging modes such as MRI to obtain a variety of information.

A key factor limiting the clinical use of PAI is the depth of penetration. PAI can image structures several centimeters deep inside tissues. This penetration depth significantly exceeds any optical imaging technique that relies on non‐scattering ballistic photons for image reconstruction, such as confocal microscopy. Compared to optical imaging techniques that rely on diffuse scattering photons, such as diffuse optical tomography, it also achieves better spatial resolution. However, conducting reliable and clinically significant imaging in bodies with depths exceeding 2–3 cm remains a major challenge. Part of the reason is that, unlike B‐mode ultrasound examination, PAI is essentially a quantitative/semi quantitative technique. The second main issue limiting the clinical application of PA is imaging contrast. Organizations naturally contain various endogenous chromophores with different but overlapping absorption spectra, such as water, HbO_2_, Hb, and other pigments. PAI can image these chromophores and even attempt to separate them using multispectral PA. Since various abnormal states can lead to changes in tissue components, such as vascular fat deposition in atherosclerotic plaque or angiogenesis in cancer development, PAI has the potential to visualize and quantify these changes compared with healthy surrounding tissues. However, relying solely on visualization of vascular and tissue oxygenation may not have sufficient clinical value for many diseases.

PAI has become an important research tool at the cellular and molecular levels by absorbing technological forces from various fields. With the continuous advancement of related technologies, it will drive biological and clinical medical research to a higher level. Nowadays, many scientists and doctors are still striving to improve this technology, pursuing higher resolution and deeper detection depth, and looking forward to a clearer view of the internal structure of the human body and real‐time monitoring of the changes in lesions. Although the widespread application of PAI in clinical imaging is still mostly in the preclinical stage, efforts are being made to translate various applications into clinical practice. With the increasing availability of clinical PAI instruments, it is expected that the use of PAI in clinical practice will significantly increase in the future.

## Conflict of Interest

The authors declare no conflict of interest.

## Author Contributions

M.H., H.Y., R.G., Y.L., X.Z., J.Z., L.F. and L.L. investigated all figures for this review and wrote the article. All authors contributed substantially to discussion of the content and reviewed and edited the manuscript before submission.
